# ECT practices in Iraq: a national audit

**DOI:** 10.1192/pb.bp.114.049247

**Published:** 2015-12

**Authors:** Nesif Alhemiary, Zainab Ali, Mohammed J. Abbas

**Affiliations:** 1 Baghdad University, Iraq; 2 Baghdad Teaching Hospital, Iraq; 3 Leicestershire Partnership NHS Trust, Leicester, UK

## Abstract

**Aims and method**

This national audit examined practice of electroconvulsive therapy (ECT) in Iraq against local standards. Data were collected by a questionnaire sent to heads of departments or medical directors in the 10 Iraqi hospitals which provide ECT and by examining case notes of all patients who had ECT in the first 6 months of 2013.

**Results**

Of the 26 psychiatric hospitals in Iraq, 10 provide ECT. There were some resource shortcomings in the ECT clinics (e.g. only 2 had a minimum of 2 rooms and all had no EEG monitoring). During the audit period, 251 patients had ECT. The mean age was 36.2 years and 51.8% were males. Bilateral ECT was used in all cases, general anaesthesia in 77.15%. The main indication for ECT was schizophrenia, followed by severe depression, resistant mania, catatonia and others.

**Clinical implications**

More work is needed to ensure all patients receive modified ECT. ECT is still used widely for schizophrenia. This needs further exploration and training.

Electroconvulsive therapy (ECT) is an effective treatment for a small number of patients who have severe mental disorders.^1,2^ Its use has declined in Western countries over the years^3^ and it has been associated with stigma.^4,5^ Unmodified ECT and its portrayal in the media might have contributed to that stigma.^6^

ECT practice varies across countries.^3,7^ In Asia, a survey of 257 institutions in 23 countries suggested that the practice may be seen as suboptimal, schizophrenia was the main indication, unmodified ECT is commonly used, electroencephalography (EEG) is not common and no formal training was given.^8^

International guidelines have been developed to ensure good practice in delivering ECT.^1,2^ In Iraq, the ECT Policy 2009 was produced by the Ministry of Health as part of a quality and standards project aimed at developing standards for care in psychiatric services. The project was carried out as a collaboration between the Royal College of Psychiatrists' Iraq Sub Committee and the Ministry of Health in Iraq using consensus conferences methodology.^9^ The aim of this audit was to assess the practice of ECT in psychiatric hospitals in Iraq by measuring the degree of compliance with the standards of the Iraqi ECT Policy 2009.

## Method

Psychiatric services in Iraq are provided in 26 hospitals. Three of these are psychiatric hospitals and the rest are psychiatry wards or units within general hospitals.^9^ We contacted medical directors or heads of psychiatry departments by email or telephone to find out how many hospitals have ECT facilities: it transpired that only 10 provide ECT treatment.

The audit was designed to investigate compliance with the Iraqi ECT Policy 2009. The policy is written in Arabic and English. We divided the standards of this policy into two groups: the first group included standards related to the requirements of ECT clinics in terms of number of rooms, equipment, medicines, processes and staff. The second group covered the standards related to individual ECT treatments. The full policy is available from authors on request.

A self-completed questionnaire covering the first group of standards and a data collection form covering the second group were developed by the authors. The questionnaire was sent by email to medical directors or heads of departments in the 10 hospitals. The data collection form was completed by psychiatry trainees (students of the Iraqi Board of Psychiatry) in these hospitals providing data from case notes of all patients who had ECT in the first 6 months of 2013. The patients were identified from the ECT clinics' records. This audit was approved by the Iraqi Board of Psychiatry.

Data were analysed with SPSS version 19.

## Results

All 10 hospitals returned the questionnaire regarding the first group of standards. For individual ECT treatments, data were available from 8 hospitals only. One hospital did not provide data and in the other no ECT treatment was given during the audit period.

Results showed that only 2 hospitals (20%) have a minimum of 2 rooms in their ECT clinics. Only 3 hospitals (30%) have an ECT machine which is less than 5 years old. No hospital has a machine which has EEG monitoring. Apart from one hospital, all machines used brief-pulse wave electrical current. Only in 4 hospitals (40%) was there an anaesthesia specialist attached to the department. There was a nurse responsible for the department in 7 out of the 10 clinics (70%). A nominated psychiatrist responsible for the department was available in 6 clinics (60%); all clinics (100%) train their ECT team and 4 (40%) conduct an annual audit of the work of the department (Table 1).

**Table 1:** Responses to the questionnaire in relation to 10 ECT clinics requirements

	*n*		
	Yes	No	No response
Anaesthesia drugs are used according to Iraqi standards	5	0	5

Is there anaesthesia specialist in the ECT department?	4	1	5

Is there is a nurse responsible for the ECT department?	7	3	0

Has a minimum of two rooms	2	8	0

ECT device has EEG	0	10	0

ECT device has different doses	8	2	0

ECT device is less than 5 years old	3	7	0

A nominated psychiatrist is responsible for the ECT department	6	4	0
If yes, was the ECT team trained by the psychiatrist?	6	0	0
If yes, did the psychiatrist conduct annual audit?	4	2	0

ECT, electroconvulsive therapy; EEG, electroencephalography.

During the audit period, 251 patients had ECT: 130 male and 121 female. The average number of patients per hospital was 26.1 (range 0-47). The mean age was 36.2 years (range 17-67 years). The majority of patients (n = 181, 72.1%) were 18 to 44 years old, followed by the age group 45 to 64 years (n = 67, 26.7%). There was only 1 adolescent patient (<18 years old) and only 2 elderly patients (>65 years old).

The gender distribution was roughly equal with 51.8% of the patients male and 48.2% female. Only about a third (28.3%) were in-patients. Written consent by the patients or their relatives was obtained in all cases. ECT was given through bilateral electrodes placement in all patients. General anaesthesia was used in 77.15% of the cases. Unmodified ECT was given in three hospitals. Further correspondence with these hospitals revealed that the reason for using unmodified ECT was the unavailability of anaesthetists.

The main indication for ECT was schizophrenia (51%), followed by severe depression (31.5%), resistant mania (10.4%) catatonia (2.4%) and others (4.4%). In those diagnosed as having schizophrenia, only 5.5% had a second opinion before ECT was prescribed and in 40% the reason was poor response to other treatments (Table 2).

**Table 2:** Demographic and clinical characteristics of the sample (*n* = 251)

	*n* (%)
Gender	
Male	130 (51.8)
Female	121 (48.2)

Service setting	
In-patient	180 (71.7)
Out-patient	71 (28.3)

ECT prescriber: psychiatric specialist	251 (100)

Diagnosis	
Severe depression	79 (31.5)
Resistant mania	26 (10.4)
Catatonia	6 (2.4)
Puerperal psychosis	1 (0.4)
Schizophrenia	128 (51)
Other	11 (4.4)

In schizophrenia, reason for ECT	
Previous good response to ECT	3 (1.2)
Poor response to other treatments	102 (40.6)
Risk to self or others	20 (8.0)
Other	3 (1.2)

In schizophrenia, second opinion was obtained: Yes	7 (5.5)

Written consent by patient or relatives: Yes	251 (100)

ECT was done under general anaesthesia: Yes	176 (70.1)

Patient was informed to fast 10 h before treatment: Yes	251 (100)

ECT dose given according to Iraqi standards: Yes	251 (100)

There was a prolonged seizure: Yes	0 (0)

Bilateral ECT: Yes	251 (100)

Patient had ECT previously: Yes	102 (40.6)

Patient notes had documentation about response to previous ECT: Yes	62 (24.7)

ECT, electroconvulsive therapy.

All of the 251 patients (100%) received a physical health examination. However, investigations were done more often in the patients who had modified ECT than those who had unmodified ECT (Table 3).

**Table 3:** Investigations

	Unmodified ECT (*n* = 75) *n* (%)	Modified ECT (*n* = 176) *n* (%)	*P*
Complete blood count	21 (28.0)	171 (97.2)	***

Fasting blood sugar	17 (22.7)	168 (95.5)	***

Urea and creatinine	8 (10.7)	170 (97.1)	***

Liver function test	8 (10.7)	171 (97.2)	***

Chest X-ray	11 (14.7)	176 (100)	***

Electrocardiogram	12 (16.0)	176 (100)	***

*** *P*<0.001, chi-squared test.

There were no statistically significant differences between males and females across clinical and demographic variables.

## Discussion

As far as we know, this is the first national audit of ECT practice in Iraq against clear and explicit standards. We collected data through two routes, a health professional questionnaire and a review of patient case notes. The audit highlighted areas of good practice and areas which need further improvement.

The majority of our patients were young (72.1% were 18 to 44 years old), which is very similar to Asian patients having ECT^8^ but different from trends in Western countries, where patients are usually elderly.^10^ Chanpattana et al^10^ suggested that this difference in age group trends could be caused by Asian population demographics and the fact that schizophrenia (with higher prevalence in younger patients) is the main indication for ECT in Asian patients. These explanations could also be valid for our Iraqi sample.

The gender distribution of our sample was roughly equal. This is slightly different from what is known in Asian countries, where more males receive ECT,^8^ and from Western countries, where more females do.^11-14^ A possible reason for this near-equal gender distribution is that, in Iraq, there was found to be no gender difference in depression;^15^ however, it is also possible that our finding was accidental.

Another finding which was very similar to Chanpattana et al's^8^ was that schizophrenia was the major indication for ECT (51% v. 41.8% in their sample). This finding is slightly different from what Iraqi psychiatrists report about the indications for ECT. In a recent survey which included 73 Iraqi psychiatrists, the first indication mentioned was depression, followed by schizophrenia.^16^ The use of ECT in schizophrenia could raise a number of questions about the appropriateness and reasons for its use. International guidelines do not recommend ECT in general cases of schizophrenia, but as an option where clozapine has already proved ineffective or intolerable.^2^ A review concluded that ETC might be an option in patients who show poor response to medication^16^ and this was also cited as the main reason for ECT in our sample. The lack or unavailability of clozapine and the difficulties associated with blood monitoring in Iraq might be one reason for poor treatment response. The practice of having a second opinion for the use of ECT in schizophrenia is still very rare in Iraq (5.5%) and needs to be encouraged.

It is encouraging that the majority (70.1%) of ECT in our study was modified ECT. This figure indicates a significant improvement in this area: although we do not have exact figures, we are aware that prior to 2003 ECT was mostly given in an unmodified way. This also seems better than the practice of ECT in Asian countries in general, where 55.7% of patients still receive unmodified ECT,^8^ but is below the 100% standard stipulated by the Iraqi ECT Policy and the practice in high-income countries.^1,2^ Unmodified ECT was applied only in three hospitals and the unavailability of anaesthetists was the only reason. Measures to address this resource issue need to be taken by the Ministry of Health. Closure of ECT clinics where general anaesthesia is not available might be one, albeit the last resort, option. In this context, we are aware that the biggest psychiatry hospital in Iraq (which has 1200 beds) has stopped ECT treatment because of the unavailability of anaesthetists and patients who need ECT are transferred to an acute hospital where a modified ECT is given. This has led to a significant reduction in the number of ECTs (Tamimy J, 2011, personal communication).

In addition to human resources, this audit identified other shortcomings such as the number of rooms, the age of the ECT machine and the lack of EEG monitoring facilities. Improving these areas could lead to an improvement in the quality of care patients receive. For example, EEG monitoring, which was absent in all clinics, could mean lower doses being given and subsequently, fewer cognitive side-effects.^18^ One way of improving these areas could be by nominating a consultant psychiatrist (in our sample, this happened in only 60% of the ECT clinics) and a nurse who are responsible for ECT delivery, oversee its practice and audit it.

Bilateral ECT was performed on all patients in compliance with the Iraqi ECT Policy, which stipulates that bilateral ECT should be used except in patients under the age of 18 (only one patient in our sample) or in elderly patients with cognitive impairment. Bilateral use of ECT seems to be the norm in many countries.^8,10,19,20^

### Limitations

One limitation of our study is that we did not collect data about the number of ECT sessions given to each patient. This information could inform us about Iraqi practice in that area, but not necessarily in measuring compliance with the policy, which does not include a standard about the number of ECT sessions. Another limitation is that we did not record how many of the consent forms were signed by patients as opposed to relatives. This could have shed light on transcultural differences in that area. In Iraq, giving consent by the family on behalf of the patient is seen as acceptable. This is something which needs to be explored further in the absence of an active mental health act. This audit has not covered ECT practice in private clinics. We know from personal contact that this is not uncommon but it is not governed by any policy. Regulations might need to be enforced to ensure good practice. We did not collect data about who administered the ECT treatments; however, we know that in Iraq ECT is administered by a psychiatry specialist or a trainee.

The recent history of Iraq has been very traumatic, with three wars, years of economic sanctions and more than 11 years of civil unrest. These major events have affected the health services', including mental health services', infrastructure. Since 2003, there have been attempts to improve and modernise mental health services in collaboration with international bodies such as the Royal College of Psychiatrists through its Iraq Sub Committee. This subcommittee has contributed to many projects,^9^ for example drafting the ECT standards. This audit has examined the practice of ECT in Iraq against these standards and identified areas for further improvement. There are resource issues that need to be addressed by the Ministry of Health and areas which could be improved by training or research. In particular, the use of ECT in schizophrenia needs further exploration.
